# Storing up Treasures: Storage Potential of *Macrolophus pygmaeus* (Hemiptera: Heteroptera: Miridae) Nymphs for Application in Biological Control

**DOI:** 10.3390/insects15060414

**Published:** 2024-06-04

**Authors:** Irina M. Pazyuk, Margarita Y. Dolgovskaya, Sergey Y. Reznik, Dmitrii L. Musolin

**Affiliations:** 1 All-Russian Institute of Plant Protection, Russian Academy of Sciences, Podbelskogo sh. 3, Pushkin, 196608 Saint Petersburg, Russia; ipazyuk@gmail.com; 2 Zoological Institute, Russian Academy of Sciences, Universitetskaya nab. 1, 199034 Saint Petersburg, Russia; bcongroup@gmail.com (M.Y.D.); reznik1952@mail.ru (S.Y.R.); 3 European and Mediterranean Plant Protection Organization (EPPO/OEPP), 21 boulevard Richard Lenoir, 75011 Paris, France

**Keywords:** cold storage, fecundity, longevity, mass rearing, predatory heteropterans, *Sitotroga cerealella*, survival, temperature

## Abstract

**Simple Summary:**

In the practice of biological control, it is often necessary to store predatory insects for a long period of time. The plant bug *Macrolophus pygmaeus* is as predatory species, mass-reared and widely used for the biological control of insect and mite pests in greenhouses. The purpose of the current study was to determine which conditions are optimal for the long-term low-temperature storage of this species and how long its nymphs can be stored. We evaluated the impact of different periods of storage (0–120 days) at different temperatures (3, 6, 9, and 12 °C) on third instar nymphs of *M. pygmaeus* (with or without access to food) on their survival, as well as on adult emergence and female fecundity. When nymphs did not have access to food, their longevity decreased with an increase in temperature during the storage period. At temperature 3 °C, the maximum longevity was about 40 days. However, at 9 °C, the longest longevity of nymphs fed on eggs of the grain moth *Sitotroga cerealella* was about 150 days. We concluded that third instar nymphs of *M. pygmaeus* fed with eggs of the grain moth could be stored at 9 °C for 30 days with a moderate (10–20%) decrease in survival and fecundity, whereas survival of starved nymphs decreased by half after 10 days of storage at 3 °C.

**Abstract:**

Long-term storage is an important component of insect mass-rearing systems, prolonging the shelf life of biocontrol agents during a low-demand period or a temporary lack of suitable food. *Macrolophus pygmaeus* is a predatory heteropteran, mass-reared and widely used for the biological control of arthropod pests in greenhouses. With the aim to determine the optimal conditions and acceptable duration of cold storage, we evaluated the impact of different periods of storage of fed and starved third instar nymphs of *M. pygmaeus* at different temperatures on nymphal survival, adult emergence, and female fecundity. Four storage temperatures (3, 6, 9, and 12 °C) were tested. The longevity of starved nymphs decreased with an increase in the storage temperature, with a maximum of about 40 days at 3 °C, whereas the longest lifetime of nymphs fed on eggs of the grain moth *Sitotroga cerealella* (about 150 days) was observed at 9 °C. Further experiments demonstrated that the third instar nymphs of *M. pygmaeus* fed with eggs of the grain moth can be stored at 9 °C for 30 days, with a moderate (10–20%) decrease in survival and fecundity, whereas the survival of starved nymphs decreased by half after 10 days of storage at 3 °C.

## 1. Introduction

Long-term storage is an important component of insect mass rearing systems. In particular, cold storage is a commonly used method to prolong the shelf life of a mass-reared biocontrol agent during a low-demand period or a temporary lack of suitable food. In addition, long-term storage allows for the accumulation of biocontrol agents for a large delivery or for a high-demand period. Although long-term cold storage is a usual element of mass production of many insects, it often exerts a number of negative effects, such as increased mortality and post-storage decrease in longevity, fecundity, and other components of the quality of biocontrol agents. That is why the development of effective cold storage methods is necessary to ensure the stable and economically effective production of many biocontrol agents [1,2,3,4,5].

Diapause is a common seasonal adaptation which allows insect to survive during various predictable adverse periods, e.g., low-temperature seasons or periods of lack of food. Usually, diapause manifests itself as a marked decrease in metabolism and a pronounced increase in resistance to unfavorable conditions [6,7,8]. Thus, diapausing individuals are often considered to be more suitable for long-term storage than actively developing ones. However, in many insects originating from the subtropical and tropical regions (which are often used for biological control of pests in greenhouses), the ability to enter diapause is weak or absent [9,10,11,12]. These species can undergo cold storage only in a state of quiescence, which is directly and immediately caused by low temperature or other unfavorable conditions [6,7,8,9]. In all cases, selection of the optimal storage protocol should be made based on the species-specific eco-physiological peculiarities of a reared biocontrol agent. Therefore laboratory studies focused on this storage potential are an essential prerequisite for the development of a cost-effective mass production of any insect proposed for use as a biocontrol agent [2,3].

Our study was conducted using *Macrolophus pygmaeus* (Rambur, 1893) (Hemiptera: Heteroptera: Miridae), a zoophytophagous predatory bug widely used for the biological control of various arthropod pests in greenhouses: whiteflies, aphids, thrips, moths (eggs and young nymphs), and spider mites [13,14,15,16,17,18]. Various biological parameters of *M. pygmaeus* have been studied in laboratories by many authors in different regions of the world [19,20,21,22,23,24,25,26,27]. In particular, it was demonstrated that *M. pygmaeus* females did not enter adult (i.e., reproductive) diapause under the different photoperiods ranging from L:D = 8:16 h to L:D = 16:8 h, i.e., under both short- and long-day conditions [22,23]. It is also known that under natural conditions, this species overwinters in the nymphal stage, although records in the literature differ: Puchkov [28,29] indicated that nymphs of the “middle instars” overwinter, whereas Cobben [30] wrote that only the last (fifth) instar nymphs can overwinter. The seasonal cycle of *M. pygmaeus* exhibiting the overwintering of nymphs was additionally, but indirectly, supported by the analysis of the collection of the Zoological Institute of the Russian Academy of Sciences in Saint Petersburg (Russia): the Heteroptera collection includes 119 specimens collected in different regions and different years, from March to November, without clear seasonal patterns of catches; the presence of adults early in the season (March and April) suggests that the species overwinter at the nymphal stage (I. Pazyuk, unpublished data). Such a seasonal cycle is an exceptional case for Miridae. Indeed, in this family, most of species hibernate in the egg stage and only some species do so as adults [30,31,32,33,34,35,36]. A small number of mirid species, in warm climates, can actively develop all year around (i.e., exhibiting a homodynamic seasonal cycle) and thus, overwinter in any stage [30,32].

In combination, these records suggest that the nymphal stage can be the optimal phase for long-term cold storage of *M. pygmaeus* in laboratories and mass production facilities. However, the storage potential of *M. pygmaeus* and in particular, the possibility of the storage of late instar nymphs for varying periods of time at different temperatures, have not been investigated, although similar studies on cold storage at different stages of development have been conducted on other species of predatory bugs [37,38,39,40,41,42,43,44,45,46,47,48].

The present study was performed to evaluate the impact of the period of storage of fed or starved third instar nymphs of *M. pygmaeus* at different temperatures on in-storage nymphal survival and post-storage fitness (adult emergence and female fecundity). Based on these data, we aimed to determine the optimal conditions and acceptable duration of cold storage of this predator.

## 2. Materials and Methods

### 2.1. Insects and General Methods

For our experiments, we used a laboratory culture of *M. pygmaeus* originated from about 20 individuals collected in 2011 in Lazarevskoe, Sochi, Krasnodar territory, Russia (43.915° N, 39.320° E). The bugs were reared in the Laboratory of Biological Plant Protection, All-Russian Institute of Plant Protection, Russian Academy of Sciences, Pushkin, Saint Petersburg, at a temperature of 24 ± 1 °C and a photoperiod of L:D = 16:8 h on tobacco plants (Virginia 202 variety). Tobacco seeds were obtained from the All-Russian Scientific Research Institute of Tobacco, Makhorka, and Tobacco Products, Krasnodar, Russia. The plants were placed in transparent 40 × 60 × 40 cm cages, covered with organza fabric. *Macrolophus pygmaeus* adults and nymphs were fed with eggs of the grain moth *Sitotroga cerealella* (Olivier, 1789), decapsulated cysts of the brine shrimp *Artemia salina* (L., 1758), and flower pollen.

### 2.2. The First Experiment: The Effect of Storage Conditions on Nymph Longevity

The aim of the first experiment was to evaluate the influence of the rearing and storage conditions on the longevity of stored *M. pygmaeus* nymphs. To start the experiment, mature females of approximately the same age were randomly selected from the laboratory culture and transferred into cylindrical plastic transparent cages, with volume of 3000 mL, covered with organza fabric. In each cage, three tomato plants (Belyi Naliv 241 cultivar) at the stage of 6–8 leaves, grown in 200 mL pots with soil, were placed in the cage, and 20 *M. pygmaeus* females were released into the container. The females were kept in these cages for one week, under the same conditions (24 ± 1 °C, L:D = 16:8 h). Then, the females were removed, and cages with tomato plants hosting laid eggs of *M. pygmaeus* were distributed among the four light and temperature regimes, i.e., combinations of low (20 °C) and high (24 °C) temperatures, with short (L:D = 10:14 h) and long (L:D = 16:8 h) photoperiods. For each of the four regimes, two cages (the progeny of 40 females) were used. Hatched nymphs of the first instar were fed three times a week (with intervals of 2 or 3 days) with eggs of the grain moth, provided in excess by spreading them on tomato leaves, and then reared under the same conditions and with the same methods to the third instar stage. This nymphal instar was selected for the storage experiments because *M. pygmaeus* reportedly overwinter in litter at this stage [28,29]. 

Randomly selected recently molted nymphs of the third instar were distributed with a soft brush among plastic Petri dishes (9 cm in diameter). In each dish, four or five nymphs were placed. Preliminary statistical analysis (see Section 3.1) demonstrated that this small variation in the rearing density did not have any significant impact on the results of the experiment. A piece of wet floral foam was placed in each dish as a source of water. Food (eggs of the grain moth glued to a piece of paper-based scotch tape, 2 × 3 cm in size) was placed in half of the Petri dishes. To maintain relative air humidity at a constant level of about 75%, the Petri dishes with the nymphs intended for storage were placed in plastic containers (24 × 16 × 14 cm) with saturated NaCl solution. For acclimatization, all Petri dishes were stored in the dark for 4 or 5 days at a temperature of 12 °C. After that, randomly selected Petri dishes were distributed among the four storage regimes: 3, 6, 9, or 12 °C in the dark. Nymphal mortality was recorded, and the floral foam was moistened three times a week, at intervals of 2 or 3 days. We considered this frequency sufficient, as the range of the studied periods was at least 15–20 times longer (up to about 40 days in starved and about 150 days in fed females). Food was replaced once a week. The experiment was continued until the death of the last nymph.

Thus, the first experiment included four factors:

1. The temperature conditions during the pre-storage development (20 or 24 °C);
2. The photoperiodic conditions during the pre-storage development (L:D = 10:14 or 16:8 h);
3. The food availability during the storage (starved or fed with the grain moth eggs);
4. The temperature conditions during the storage (3, 6, 9, or 12 °C).

In combination, these four factors resulted in 32 experimental treatments, and for each treatment, two or three Petri dishes were used (with four or five nymphs per Petri dish). Nymphal longevity (from the beginning of storage to death) was a single parameter measured in the first experiment. To avoid pseudoreplicates, instead of an individual insect, one Petri dish was used as a unit for statistical analysis. With this aim, the mean longevity of all nymphs in each Petri dish was calculated.

### 2.3. The Second Experiment: The Effect of Storage Conditions on Nymphal Survival and Female Fecundity

The aim of the second experiment was to evaluate the influence of storage, not only on nymphal survival, but also on female fecundity, i.e., on the two important parameters strongly influencing the rate of population increase. To start this experiment, we used the same method as that used in the first experiment. Pre-storage nymphal development occurred at a temperature of 20 °C and L:D = 10:14 h. The cold storage conditions (3 °C for starved and 9 °C for fed nymphs) were chosen based on the results of the first experiment, i.e., at these temperatures, the highest nymphal longevity of the starved and fed nymphs was observed. In contrast to the first experiment, nymphs were stored not until their death, but for a certain period of time: 10, 20, or 30 days for starved nymphs kept at 3 °C and 30, 60, 90, or 120 days for fed nymphs kept at 9 °C. Food (eggs of the grain moth) and water were provided to the stored nymphs following the same protocol as that used in the first experiment, except that food was replaced once a week. For acclimatization, before and after the storage, the nymphs were kept for 4 or 5 days in the dark at 15 °C. At the beginning of the experiment, five nymphs were placed in each Petri dish. After the storage and acclimatization, all survived nymphs were transferred to the optimal photo-thermal conditions (temperature 24 °C, L:D = 16:8 h) to complete their nymphal development. 

During the post-storage period, nymphs were kept on tomato seedlings (Belyi Naliv 241 cultivar) at the 2–4 leaf stage. All surviving nymphs from one Petri dish were placed on each plant, which means from one to five nymphs per plant, depending on the mortality during the storage. Preliminary statistical analysis (see Section 3.2) demonstrated that the number of nymphs per plant did not have any significant impact on the results of the experiment. The plants were grown in small (100 mL) plastic pots with soil; these small pots were placed in larger (500 mL) plastic containers, covered with cotton cloth. During the post-storage period, nymphs were fed with eggs of the grain moth, provided two or three times a week in excess. Eggs of the grain moth were glued with non-toxic polyvinyl glue to hard paper cards (2 × 3 cm in size) placed on the soil near the tomato plants. Emerging adults were split into female and male pairs. When possible, males developed from nymphs stored during the same period at the same temperature were used; otherwise, males were taken from other treatments or from the laboratory culture. The pairs of adults were placed in containers of the same size (500 mL) holding small tomato plants. They were fed using the same protocol and kept for 10 days under the same conditions as the post-storage nymphs. If a female died during this period, the data obtained by the date of death were recorded and used for statistical analysis. Dead males were not replaced because it is known that *M. pygmaeus* females receive enough sperm during only one mating event to fertilize most of their eggs during the next 20 + days [49]. At the end of the 10-day-long test period, *M. pygmaeus* adults were removed, and tomato plants with eggs laid on them were kept under the same photo-thermal conditions for an additional 12–14 days, until the nymphs began to hatch. Then, the hatched nymphs of the first instar were counted and removed at intervals of 2 or 3 days over a period of about 10 days (until the end of the nymphal hatching). After that, the total number of hatched nymphs was used as an estimate of the number of eggs produced by a female during the first 10 days of her adult life, which in turn, was used as an estimate of the female’s potential fecundity. In addition to the experimental (stored) nymphs, some randomly selected nymphs of the third instar were used as a control for the fecundity test: instead of being placed in storage, they were transferred to the same optimal conditions (temperature 24 °C, L:D = 16:8 h) and reared to the adult stage following the same protocol as that followed for the experimental individuals.

Thus, the second experiment included two factors:

1. The storage conditions (3 °C without feeding or 9 °C with feeding);
2. The period of storage (0, 10, 20, or 30 days at 3 °C and 0, 30, 60, 90, or 120 days at 9 °C).

In combination, the second experiment included eight regimes: seven experimental treatments (three periods of storage at 3 °C and four periods of storage at 9 °C) and the control. To avoid pseudoreplicates, instead of an individual insect, one Petri dish was used as a unit for statistical analysis. With this aim, the percentage of survived nymphs and the mean fecundity of emerged females were calculated for each Petri dish. For each treatment, 35–47 Petri dishes were used, and five nymphs were placed in each Petri dish for storage.

Based on the data obtained in the second experiment, the following two parameters were determined:

1. The nymphal survival (i.e., the proportion of nymphs that reached the adult stage);
2. The fecundity (i.e., the number of eggs laid by a female during the first 10 days of her adult life).

### 2.4. Statstical Analysis

The data were analyzed using SYSTAT 10.2 (Systat Software Inc., Richmond, VA, USA). The data (nymphal longevity, nymphal survival, and female fecundity) were not normally distributed and therefore, were ranked and then evaluated using GLM analysis, ANOVA, and the Tukey HSD test.

## 3. Results

### 3.1. The First Experiment: Effect of Storage Conditions on Nymph Longevity

Most of the *M. pygmaeus* nymphs used in the first experiment died after a certain period of storage, but 11% of fed nymphs stored at 9 °C, and 85% of fed nymphs stored at 12 °C reached the adult stage. For these nymphs, the period from the beginning of the third instar to the adult molt was 107 ± 5 and 43 ± 8 days at 9 and 12 °C, correspondingly (here and below, means and SD are provided). These nymphs were excluded from further analysis. Four-way ANOVA of the ranked data for the rest of nymphs (i.e., the nymphs which died during storage) demonstrated that the longevity of stored nymphs highly significantly depended on the temperature experienced and the food availability during storage (Table 1). The influence of the photoperiod and temperature conditions during pre-storage development was not significant. Regarding pairwise interactions, only two of these (food availability with temperatures during the nymphal development and during the storage) were highly statistically significant (Table 1). Therefore, the data for two pre-storage photoperiods and two pre-storage temperatures were pooled for further analysis. As was noted above in the Section 3.2, in one Petri dish, either four or five nymphs were placed for the storage period, and this variation could also have had some effect on longevity. To reveal this effect, GLM analysis was conducted, which supported the conclusions which had been made based on ANOVA and demonstrated that the influence of the rearing density (i.e., the initial number of nymphs in a Petri dish) on nymphal longevity during the storage period was not statistically significant (Table 2).

**Figure 1:**
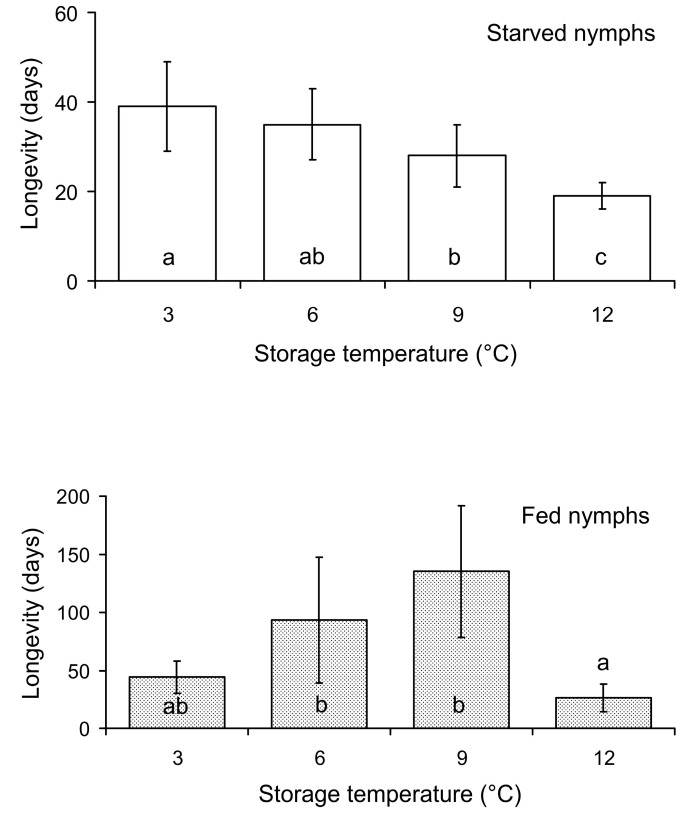
Longevity of starved and fed *Macrolophus pygmaeus* nymphs in relation to the storage temperature. Means and SD are shown; *n* = 4–11 Petri dishes per bar. Bars of the same graph labeled with different letters are significantly different (*p* < 0.05 by the Tukey HSD test on ranked data).

As could be expected, the fed nymphs survived much longer than the starved individuals (compare top and bottom graphs in Figure 1). In addition, the longevity of the starved nymphs decreased with the increase in the storage temperature, with a maximum of about 40 days at 3 °C, whereas the highest longevity of the fed nymphs (about 150 days) was observed at 9 °C (Figure 1). Based on these results, the second experiment was conducted with starved nymphs that were stored at 3 °C and fed nymphs that were stored at 9 °C.

### 3.2. The Second Experiment: Effect of Storage Conditions on Nymphal Survival and Female Fecundity

**Figure 2:**
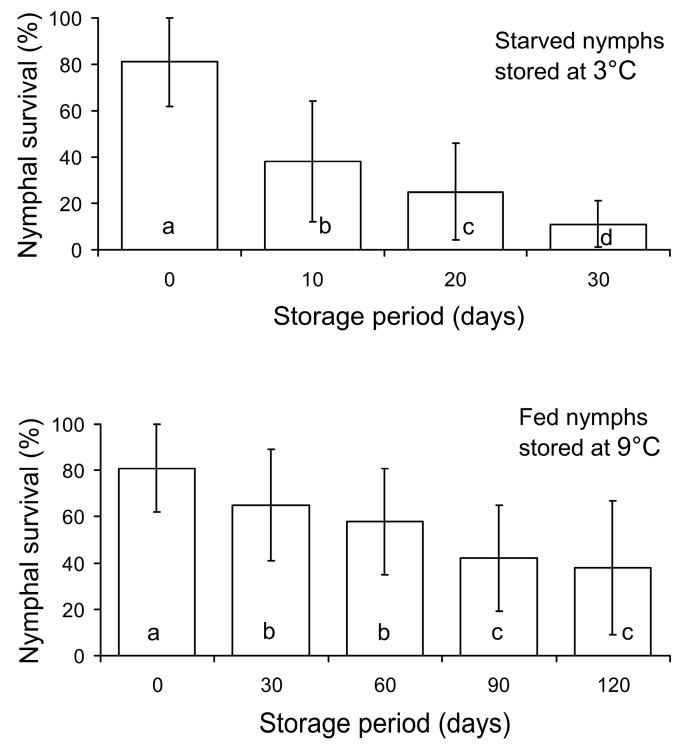
Survival of *Macrolophus pygmaeus* nymphs (i.e., the proportion of nymphs that reached the adult stage) in relation to the storage period and temperature. Means and SD are shown; *n* = 35–47 Petri dishes per bar. Bars of the same graph labeled with different letters are significantly different (*p* < 0.05 by the Tukey HSD test).

The one-way ANOVA of the results of the second experiment demonstrated that nymphal survival highly significantly depended on the duration of the storage period, both in starved nymphs stored at 3 °C (*F* = 73.5, *n* = 176, *df* = 3, *df*_error_ = 172, *p* < 0.001) and in fed nymphs stored at 9 °C (*F* = 20.3, *n* = 220, *df* = 4, *df*_error_ = 215, *p* < 0.001). As can be seen in Figure 2, nymphal survival (i.e., the proportion of nymphs that reached the adult stage) gradually decreased to about 10% in the starved nymphs stored for 30 days at 3 °C and to about 40% in the fed nymphs stored for 120 days at 9 °C. Considering that nymphal survival of the controls was about 80%, we conclude that 10 days of storage of the starved nymphs at 3 °C caused an approximately 50% decrease in nymphal survival, whereas nymphal survival of the fed nymphs stored at 9 °C decreased by about 20% after 30 days of storage, by about 30% after 60 days of storage, and by about 50% after 90 and 120 days of storage (Figure 2).

**Figure 3:**
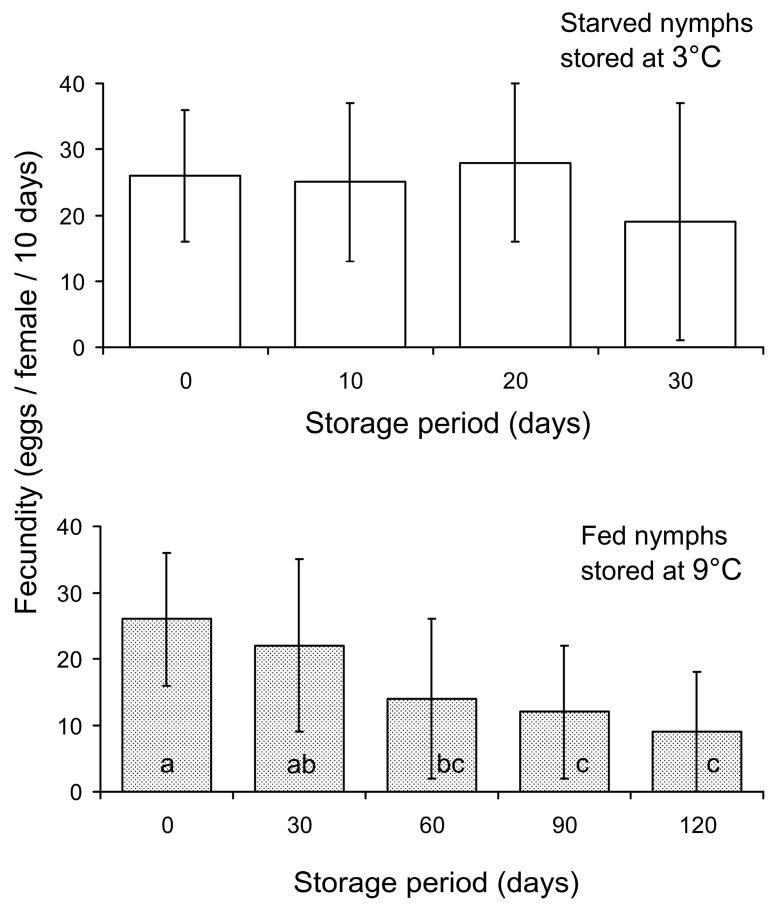
Fecundity of *Macrolophus pygmaeus* females (the number of eggs laid by a female during the first 10 days of her adult life) in relation to the storage period and temperature exposure as nymphs. Means and SD are shown; *n* = 9–31 Petri dish per bar. Bars of the same graph labeled with different letters are significantly different (*p* < 0.05 by the Tukey HSD test on ranked data); the absence of letters means the absence of any significant pairwise difference.

As indicated by ANOVA, fecundity (i.e., the number of eggs laid during the 10-day test period) of *M. pygmaeus* females also highly significantly decreased with the increase in the duration of storage of the fed nymphs at 9 °C (*F* = 10.0, *n* = 134, *df* = 4, *df*_error_ = 129, *p* < 0.001). However, when starved nymphs were stored at 3 °C, ANOVA demonstrated that the fecundity of the resulted females did not significantly change with the increase in the duration of storage (*F* = 1.3, *n* = 75, *df* = 3, *df*_error_ = 71, *p* = 0.285). As the number of nymphs placed on one plant for the period of post-storage development was not constant, this factor can also influence the fecundity of the resulted females. To reveal this effect, GLM analysis was used. When starving nymphs were stored at 3 °C (*n* = 75), the fecundity of the females was not significantly dependent on either the duration of storage (*C* = −0.719 ± 0.801, *p* = 0.373; here and below, the coefficient of regression ± SE and *p*-value are provided) or the rearing density (i.e., the number of nymphs per plant; *C* = −14.9 ± 31.6, *p* = 0.638). When fed nymphs were stored at 9 °C (*n* = 134), the fecundity of the females highly significantly depended on the duration of storage (*C* = −0.621 ± 0.106, *p* < 0.001) but not on the number of nymphs per plant (*C* = −12.1 ± 18.7, *p* = 0.519). Indeed, as can be seen in Figure 3, the fecundity of the females developed from the nymphs stored at 9 °C gradually decreased with the storage period and dropped to about 30% of that in the controls when the nymphs were stored for 120 days, whereas a decrease in fecundity observed in the starved females after 30 days of storage at 3 °C was not statistically significant. Regarding shorter storage periods, the mean fecundity of *M. pygmaeus* females decreased by approximately 15% after 30 days and by about 50% after 60 days of storage of the fed nymphs at 9 °C.

## 4. Discussion

Two experiments demonstrated that fed *M. pygmaeus* nymphs of the third instar can be stored for 30 days at 9 °C with a moderate (10–20%) decrease in both nymph survival and female fecundity, whereas 60 days of storage at 9 °C caused a rather high (40–50%) decrease in these parameters (Figure 2 and Figure 3). The storage potential of the starved nymphs was much lower: a 50% decrease in survival was already observed after 10 days of storage at 3 °C (Figure 2), although the fecundity of the females developed from the survived nymphs was not lower than that of the non-stored controls (Figure 3). The storage potential of other predatory bug species studied in this respect varied widely between species, sexes, and stages of development. Thus, storage at low temperatures reduced fecundity in some species [38,43], but not in others [44,48]. The starvation of adults during low temperature storage resulted in reduced survival [37], but longevity was greater in females than males [37,41]. The storage potential varied among developmental stages in some species [1,39,40], but the differences were minimal in others [46,48]. The alternation of storage temperature resulted in a higher survival rate [45]. Storage at higher temperature was only possible under short-day conditions [47].

## 5. Conclusions

For practical applications, we conclude that the third instar nymphs of *M. pygmaeus* fed with eggs of the grain moth can be stored at 9 °C for 30 days, with a moderate (10–20%) decrease in survival and fecundity, whereas the survival of the starved nymphs decreased by half after 10 days of storage at 3 °C.

## Figures and Tables

**Table 1:** Significance of the influence of photoperiodic and temperature conditions of the pre-storage development, storage temperature, and feeding during the storage on the longevity of the stored *Macrolophus pygmaeus* nymphs.

A Factor or a Combination of Two Factors	The Results of Four-Way ANOVA on Ranked Data (*n* = 68, *df*_error_ = 36): The Number of Degrees of Freedom (*df*), Fisher Coefficient (*F*), and *p*-Value
Temperature during development	*df* = 1, *F* = 2.8, *p* = 0.103
Photoperiod during development	*df* = 1, *F* = 1.3, *p* = 0.254
Temperature during storage	*df* = 3, *F* = 18.9, *p* < 0.001
Feeding during storage	*df* = 1, *F* = 52.7, *p* < 0.001
Temperature during development × Photoperiod during development	*df* = 1, *F* = 3.1, *p* = 0.088
Temperature during development × Temperature during storage	*df* = 3, *F* = 0.7, *p* = 0.566
Temperature during development × Feeding during storage	*df* = 1, *F* = 8.9, *p* = 0.005
Photoperiod during development × Temperature during storage	*df* = 3, *F* = 3.3, *p* = 0.032
Photoperiod during development × Feeding during storage	*df* = 3, *F* = 5.3, *p* = 0.027
Temperature during storage × Feeding during storage	*df* = 3, *F* = 8.7, *p* < 0.001

**Table 2:** Influence of photoperiodic and temperature conditions of the pre-storage development, storage temperature, feeding during the storage, and initial number of nymphs placed in one Petri dish on the longevity of the stored *Macrolophus pygmaeus* nymphs.

Factors	The Results of GLM Analysis on Ranked Data (*n* = 68): The Coefficient of Regression ± SE and *p*-Value
Temperature during development	*C* = –0.684 ± 0.988, *p* = 0.491
Photoperiod during development	*C* = –0.923 ± 0.661, *p* = 0.167
Temperature during storage	*C* = –2.197 ± 0.646, *p* = 0.001
Feeding during storage	*C* = –4.079 ± 0.527, *p* < 0.001
The number of nymphs per Petri dish	*C* = –7.172 ± 4.783, *p* = 0.139

## Data Availability

Data are available upon email request to the corresponding author.
